# Functional T Cell Reactivity to Melanocyte Antigens Is Lost during the Progression of Malignant Melanoma, but Is Restored by Immunization

**DOI:** 10.3390/cancers13020223

**Published:** 2021-01-09

**Authors:** Anna Przybyla, Alexander A. Lehmann, Ting Zhang, Jacek Mackiewicz, Łukasz Galus, Greg A. Kirchenbaum, Andrzej Mackiewicz, Paul V. Lehmann

**Affiliations:** 1 Research and Development Department, Cellular Technology Limited (CTL), Shaker Heights, OH 44122, USA; przybyla.anna.ump@gmail.com (A.P.); alexander.lehmann@immunospot.com (A.A.L.); ting.zhang@immunospot.com (T.Z.); greg.kirchenbaum@immunospot.com (G.A.K.); 2 Department of Cancer Immunology, Medical Biotechnology, Poznan University of Medical Sciences, 61-866 Poznan, Poland; mackiewicz.aa@gmail.com; 3 Department of Medical and Experimental Oncology, University of Medical Sciences, 60-355 Poznan, Poland; jmackiewicz@ump.edu.pl (J.M.); lukasz_galus@wp.pl (Ł.G.); 4 Department of Diagnostics and Cancer Immunology, Greater Poland Cancer Center, 61-866 Poznan, Poland; 5 Chemotherapy Department, Greater Poland Cancer Center, 61-866 Poznan, Poland

**Keywords:** melanoma, genetic whole-cell therapeutic melanoma vaccine (AGI-101H), CD8+ T cells, melanoma antigens, ELISPOT

## Abstract

**Simple Summary:**

Healthy humans develop spontaneous CD8+ T cell responses to melanoma associated antigens (MA) expressed by normal melanocytes. This natural autoimmunity directed against melanocytes might confer protection against the development of malignant melanoma (MM), where MA are overexpressed tumor-associated antigens. We report that functional T cell reactivity to MA is diminished in untreated MM patients. Three lines of evidence suggest that the MA-reactive T cells present in healthy subjects undergo exhaustion once MM establishes itself. First, only the MA-specific T cell reactivity was affected in the MM patients. Second, in these patients, the residual MA-specific T cells were functionally impaired, showing a diminished per cell IFN-γ productivity. Third, immunizations with allogeneic melanoma cells restored natural CD8+ T cell autoimmunity to MA.

**Abstract:**

Healthy human subjects develop spontaneous CD8+ T cell responses to melanoma associated antigens (MA) expressed by normal melanocytes, such as Tyrosinase, MAGE-A3, Melan/Mart-1, gp100, and NY-ESO-1. This natural autoimmunity directed against melanocytes might confer protection against the development of malignant melanoma (MM), where MA are present as overexpressed tumor-associated antigens. Consistent with this notion we report here that functional T cell reactivity to MA was found to be significantly diminished to MAGE-A3, Melan-A/Mart-1, and gp100 in untreated MM patients. Three lines of evidence suggest that the MA-reactive T cells present in healthy subjects undergo exhaustion once MM establishes itself. First, only the MA-specific T cell reactivity was affected in the MM patients; that to third party recall antigens was not. Second, in these patients, the residual MA-specific T cells, unlike third party antigen reactive T cells, were functionally impaired, showing a diminished per cell IFN-γ productivity. Third, we show that immunization with MA restored natural CD8+ T cell autoimmunity to MA in 85% of the MM patients. The role of natural T cell autoimmunity to tumor-associated MA is discussed based on discrete levels of T cell activation thresholds.

## 1. Introduction

Among all cancers, T cell responses to melanoma are arguably the best characterized, yet much is still to be learned about the basic rules that govern the induction and maintenance of functional CD8+ effector T cells vs. the development of various forms of T cell tolerance. It is well established that melanoma-specific CD8+ T cells can be detected in melanoma patients (MP) after the tumor has metastasized [1]. Melanoma is thought to be among the most immunogenic tumors due to its exceptionally high UV-driven mutational burden, allowing for the creation of unique neoantigens recognizable to T cells [2]. While the initial T cell response is likely to target these mutated “foreign” tumor-specific antigens, the anti-tumor T cell response apparently spreads [3,4,5,6,7], to involve unmodified “self” antigens, so-called melanoma-associated antigens (MA). The latter can be classified as melanocyte lineage-specific antigens (MART-1/Melan-A, Tyrosinase, gp100) and antigens derived from genes expressed in testis and a variety of cancers (including MAGE-family, NY-ESO-1) [8]. CD8+ T-cell immunity directed against such MA is arguably the best-studied form of tumor-specific T-cell immunity in humans. Based on MHC multimer measurements, MA-specific CD8+ T cells can be detected in MP sometimes in substantial numbers [9], but their abundance is not accompanied by a clinically significant antitumor effect [10]. This apparently counter-intuitive finding can be reconciled with the notion that MA-specific CD8+ T cells in MP continuously progress from an early effector “transitional” into a dysfunctional T cell state with eventually dysfunctional T cells prevailing [11]. Thus, MA-specific CD8+ T cells are present, but become exhausted due to persistent antigen stimulation [12]. Such exhausted T cells are characterized by the loss of classical CD8+ T cell effector functions, including their ability to kill, and to produce pro-inflammatory cytokines such as interferon-gamma (IFN-γ) [13,14], resulting in part from their overexpression of inhibitory receptors such as programmed cell death (PD)-1 [15]. One of the major goals of cancer therapy is to restore tumor-specific CD8+ T cell effector functions by overcoming T cell exhaustion [16].

Most insights on MA-specific CD8+ T cells in MP have been gained using MHC multimers (tetramers/pentamers/dextramers). While these constructs are ideal for revealing the numbers of CD8+ T cells that are specific for a given MA peptide, along with the phenotype of the peptide-specific T cells, they are less suited to directly detect the actual functionality of such CD8+ T cells [17]. Exhausted CD8 T cells, however, comprise of heterogeneous cell populations with unique differentiation and functional states [13]. Because CD8+ T exhaustion is imprinted at an epigenetic level, it is inherited to daughter cells. Thus, temporary/partial therapeutic restoration of CD8+ T cells functions can occur in exhausted CD8+ T cells without fully reverting the phenotype; CD8 T cells may need to be epigenetically reprogrammed to achieve long-lasting reinvigoration [18]. Moreover, the multimer approach inherently is likely to cover only a fraction of the antigen-specific CD8+ T cell repertoire because CD8+ T cell responses can be restricted by all MHC class I molecules present in an individual, and they can recognize multiple epitopes on a given antigen [19]. Last but not least, multimers lack the sensitivity to reliably detect CD8+ T cell populations that occur in very low frequency, such as MA-specific CD8+ T cells in healthy individuals [20]. The functional MA-specific functional CD8+ T cell repertoire in healthy subjects, therefore, remained largely unexplored.

Relying on an ELISPOT approach that detects functional CD8+ T cells even as rare as 1:250,000 peripheral blood mononuclear cells (PBMC), and using mega peptide pools that cover all epitopes on MA, we have shown that in healthy human donors (HD) primed MA-specific CD8+ T cells can be detected [21]. Therefore, apparently not only melanoma cells are immunogenic after they have metastasized, but already regular melanocytes can trigger tumor-associated antigen-specific T cell responses, and doing so without evidently harming regular melanocytes. In analogy to natural autoantibodies [22], which also should be forbidden based on the self/non-self-model, we called these CD8+ T cells natural autoreactive T cells. These MA-specific T cells recognize tyrosinase, MAGE-A3, melan/Mart-1, gp100, and NY-ESO-1 [21]. Reminiscent of anti-viral T cell responses [19], the T cell response against these MA is aleatory [23], i.e., it is directed against different MA in different individuals [21]. These MA-specific natural CD8+ T cells are in a resting memory cell stage at their isolation from the body, but within 3 days of antigen re-stimulation, they become cytolytic effector cells as resting memory cells generally do.

It can be assumed that such naturally autoreactive CD8+ T cells get primed during sunburns we suffer when melanocyte activation occurs in an inflamed skin, and thus both requirements for priming of a T cell response are warranted: the temporary overexpression of the melanocyte antigens providing increased levels of TCR ligand sufficient to exceed the activation threshold of low-affinity CD8 cells [24], and the “danger” environment required for the induction of immunity vs. of tolerance [25]. While such naturally autoreactive melanocyte-specific T cells might actively enhance the inflammatory response associated with the sunburn, and as such might in the long term promote melanoma development, in healthy hosts, they apparently become negligent of the autoantigen as soon as its expression level returns to normal as these cells are resting CD8+ T memory cells at their isolation [21]. It remains to be established whether such naturally autoreactive CD8+ T cells represent a novel line of immunity protecting healthy subjects from developing melanoma.

In this communication, we have addressed the question of what happens to such fully functional naturally autoreactive MA-specific CD8+ T cells when melanoma develops. Overexpressed MA are now present in the host and thus should re-engage these T cells. The data presented in the following suggest that instead of being recruited into the anti-tumor effector cell pool, these T cells become functionally silenced.

## 2. Results

### 2.1. Natural T Cell Reactivity to Melanocyte Antigens in Healthy Donors

**Figure 1:**
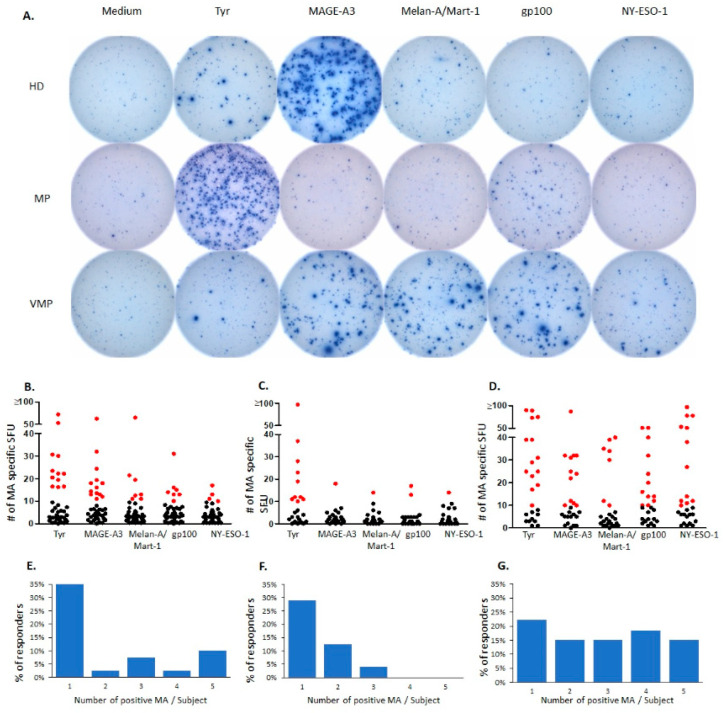
IFN-γ ELISPOT recall responses to melanocyte antigens. (**A**), Representative well images (96-well plate). The melanocyte antigens (MA) specified across the top were tested on PBMC of healthy donors (HD), untreated melanoma patients (MP), and MP vaccinated with AGI-101H (VMP). Well images are shown for a subject representative of each cohort. (**B**–**D**): The number of MA-specific CD8+ T cells in PBMC of 40 HD (**B**), 24 MP (**C**), and 27 VMP (**D**). For each subject, the IFN-γ ELISPOT recall response was tested after exposing the PBMC to the melanocyte antigens specified on the X-axis. Each data point represents the mean SFU count established in 250,000 PBMC/well, in triplicate wells. Positive T cell responses, as defined in Materials and Methods, are highlighted in red. (**E**–**G**): The number of MA eliciting positive T cell responses in 40 HD (**E**), 24 MP (**F**), and 27 VMP. PBMCs of each subject were tested in an IFN-γ ELISPOT assay for T cell reactivity to the five MA: Tyrosinase, MAGE-A3, Melan-A/Mart-1, gp100, and NY-ESO-1. The number of MA that elicited a positive response per donor (X-axis) is shown vs. the percentage of subjects in each cohort responding to that number of MA, specified on the Y-axis.

As we reported before, healthy human donors (HD) frequently exhibit melanoma associated antigen- (MA-) specific functional CD8+ T cells [21]. An example of such a recall response is provided in Figure 1A, showing an HD who gave a dominant recall response to MAGE-A3, and responded in a subdominant fashion to Tyrosinase, also a MA. In this HD, the frequency of T cells responding to other MAs, namely melan-A/mart-1, gp100, and NY-ESO-1 were below the detection limit of the assay as performed, that is, indistinguishable from the medium control. Occasional subjects of the HD cohort (*n* = 40) responded to the latter MA as well (Figure 1B). Recall responses to at least one MA were detected in 23 of 40 (57.5%) HD. As shown in Figure 1F, the prevalent response type in HDs targeted a single MA (seen in 14 of 40 HD, 35%), whereas all five MA tested positive in four HDs in our cohort (10%). Two and four MAs were recognized by one HD each (2.5%) and three of the 40 HD responded to three MAs. This MA-reactivity profile in the HD cohort serves as the reference against which we compared MA-reactivity in unvaccinated and vaccinated melanoma patients.

### 2.2. Natural Melanocyte Antigen-Specific T Cell Immunity Is Deficient in Patients with Untreated Malignant Melanoma

We tested 24 melanoma patients (MP, all Stage IV, and pharmacologically untreated) for their recall responses to MA. In this cohort, the MA-triggered recall responses were largely reduced compared to HC. As seen in Figure 1F, seven of the 24 (29%) MP responded to a single MA. The MA-induced SFU counts were lower in MP than in HC (Figure 1C vs. Figure 1B). In particular, T cell reactivity was significantly diminished to MAGE-A3, Melan-A/Mart-1, and gp 100 in MP vs. HC (see Figure S1). Additionally, the MA-triggered SFU size was decreased in the MP vs. the SFU sizes seen in HC (see in Figure 1A, MP’s response to gp100 vs. the HC’s response to MAGE-A3). Reduced SFU sizes reveal impaired per T cell IFN-γ secretion [26] being a characteristic of T cells that have developed partial anergy [14]. These changes in MP’s T cell responses were MA-specific as the MP’s T cell recall response to third party recall antigens, CEF peptides, was not impaired compared to the HC cohort (Figure S1F). The CEF peptide pool consists of immune dominant epitopes of Cytomegalo-, Epstein Barr- and Flu Virus [27].

### 2.3. Successful Vaccination Restores Natural T Cell Autoimmunity in Melanoma Patients

AGI-101H is a melanoma vaccine that relies on super-IL-6 transfected allogeneic melanoma cells [28]. In phase 2 clinical trials AGI-101H was shown to have a 50% response rate on Stage III and Stage IV melanoma patients with those responding surviving up to 15 years [29,30,31]. Two years after the trial ended, we tested 27 clinically stable long-term survivors who continued to receive every month AGI-101H injections as maintenance treatment. Therefore, the AGI-101H vaccinated melanoma patients (VMP) tested in this study constitute a homogenous group of stable long-term survivors who had been hyper-immunized with MA due to the repeated injections of the allogeneic melanoma cells. Twenty-three of 27 (85%) subjects in the VMP cohort responded to MA with SFU response magnitudes of MA-specific T cells equal to or elevated compared to that in HD (Figure 1B vs. Figure 1D, and Supplemental Figure S1). Statistical analysis of these data comparing the VMA cohort with the unvaccinated MP group showed a highly significant increase in the numbers of IFN-γ producing T cells specific for all five MA (see Supplemental Figure S1). These VMP subjects commonly targeted several MA (Figure 1D): four of the 27 VMP (14.8%) responded to all five MA tested, five displayed T cell responses to four MA (18.5%), four (14.8%) responded to three MA, dual MA recognition was seen also in four subjects (14.8%) while single MA recognition occurred in 22% of the VMP (6 of 27). Original data for a representative VMP are shown in Figure 1A. This subject displayed strong recall responses to MAGE-A3, melan/mart-1, and gp100 and weaker responses to Ny-Eso-1 and tyrosinase. The described changes in VMP’s vs. MP’s T cell responses to MA were MA-specific because there was no significant difference in the two cohort’s T cell response to third party antigens consisting of CEF peptides (Figure S1F). CEF consists of immune dominant peptides of cytomegalo-, Epstein Barr- and influenza virus [27]. Hyper-immunization with IL-6 transfected allogeneic melanoma cells therefore not only successfully restored T cell reactivity to MA in MP, but raised it beyond the level present in HD.

## 3. Discussion

The first set of data presented here establish that in untreated MP natural T cell reactivity to MA is severely reduced. A similar observation was reported for breast cancer: HD were shown to respond to the breast cancer-associated antigen HER-2, but breast cancer patients selectively lacked this reactivity [32]. At this point, there are two possible interpretations for the lack of natural T cell autoreactivity in cancer patients. One is that pre-existing autoimmunity protects against cancer development, and subsequently, in cancer patients, the non-responder phenotype is prevalent. An alternative and equally plausible interpretation for the lack of natural T cell autoreactivity in melanoma (and breast cancer) patients is the selective inactivation of these T cells: the persisting tumor will continue to stimulate and eventually exhaust them.

Beyond establishing the fact of absent natural T cell autoreactivity in melanoma, our data do not permit us to distinguish between the above two possibilities. The first interpretation is simple and may not merit in-depth discussion. Accordingly, individuals lacking natural anti-MA T cell immunity have developed melanoma and their immunization with an allogeneic melanoma vaccine induces protective anti-MA immunity. As this vaccine is allogenic, but we used a self-restricted detection system, cross-presentation must have triggered the anti-MA T cell response detected in the VMP subjects. However attractive, this first hypothesis is due to its simplicity, the data can also be interpreted in the context of multiple discrete T cell activation threshold levels operating in the three cohorts, differentially affecting fates of T cells based on their respective affinity for MA. Because of its complexity, the latter may merit more deliberation.

Tumor-associated antigens like the MA we studied here fall in the realm of T cell autoimmunity. They are normal self-proteins recognized or neglected by T cells based on differential expression levels and microenvironments. In the field of basic T cell autoimmunity activation threshold-based repertoire selections and exhaustions are well-established concepts on which we build the tiered threshold hypothesis model discussed in the following. It can be deduced from well-defined elements. One is the consensus view in the field of autoimmunity about the rules that govern the induction of a productive T cell response vs. various types of abortive activation states that result in T cell tolerance, as summarized in [33]. Such knowledge primarily emerged by studies of fates of autoreactive T cell receptor-(TCR) transgenic T cells, and thus applies to T cells of a single discrete affinity level for the self-antigen. The second component of the tiered threshold hypothesis is based on the assumption that the T cell repertoire that is specific for most antigens (including autoantigens and tumor-associated antigens, i.e., MA as well) comprises of a spectrum of clones ranging from high to low affinity [34], each being activated at a discrete activation threshold level [24]. It follows that T cells with specificity for an autoantigen/tumor antigen do not have uniform destinies, but their respective affinities for that antigen predisposes them to different fates and contributions to anti-tumor immunity. The third component builds on the consensus view on T cells that exhaust due to the persistence of antigen, as summarized in [18]. From these basic building blocks, our discussion below logically flows.

T cell activation occurs when a signaling threshold is reached that is primarily dictated by the extent of the T cell receptors (TCR) ligation [24]. In reaching this threshold, the TCR’s affinity for the TCR-ligand (TCR-L, consisting of the nominal antigen peptide “X” presented on an MHC molecule) is reciprocally related to the TCR-L density on the antigen-presenting cell (APC): T cell clones with high-affinity TCR for TCR-L X will become activated even at low densities of TCR-LX on the APC, while T cells with a TCR that has low affinity for TCR-LX need TR-LX to be present in high density [34]. In the context of autoantigen (and hence tumor-associated antigen) recognition by T cells, it is primarily the autoantigen expression level that defines the density of TCR-LX on the APC, thus explaining whether a T cells activation threshold is reached.

In the case of MA-specific T cells, the first discrete TCR-L threshold level (“Level 1”) is set by MA’s constitutive expression in the thymus and on resting melanocytes. During negative selection, the MA-specific T cell repertoire is trimmed off clones whose TCR is of high enough affinity to become activated by the Level-1 antigen presentation threshold, while MA-specific clones of lower affinity surviving as naïve T cells that are “ignorant” of the autoantigen [33]. Precursor cells for MA-specific effector T cells should fall in this > Level 1 TCR affinity category. 

During sunburns, the skin becomes inflamed and melanocytes become mobilized now expressing MA at a higher level than resting melanocytes. This results in an increased TCR-L level on activated melanocytes (Level-2): T cell clones whose TCR affinity for MA is high enough to reach the activation threshold by Level-2 TCR-L becomes primed effector/memory T cells. We assume that such T cells represent the naturally autoreactive CD8+ T cells we have observed in HD. These T cells can target inflamed melanocytes, but they cannot attack resting melanocytes as TCR-L density on the latter is subthreshold for them, and as such, they pose no autoimmune threat. Once the skin inflammation subsides, TCR-L expression again settles in at Level 1, and the naturally autoreactive T cells, now memory cells, become ignorant of the autoantigen. Subsequently, the naturally autoreactive CD8 T cells in HD do not undergo exhaustion. Matching this hypothesis, at isolation, they were found to be fully functional, resting memory cells, being IFN-γ positive, granzyme B negative [21]. 

MAs are overexpressed in melanoma cells compared to melanocytes [35], resulting in Level 3 TCR-L density. Exceeding their > Level 1 activation threshold, naturally autoreactive CD8 cells will become stimulated by melanoma cells. Such T cells might kill off melanoma cells and protect from melanoma development altogether. If the tumor persists, after initially combatting the tumor, these naturally autoreactive T cells are likely to be driven by the persisting antigen stimulation into a state of partially reversible unresponsiveness (anergy) and eventually into exhaustion and senescence [14]. Recently it has also been shown that a large population of melanoma-infiltrating CD8 T cells shows continuous progression from an early effector into a dysfunctional T cell state suggesting that functional silencing occurs rapidly in the tumor itself [11]. Subsequently, the naturally autoreactive T cells become undetectable in patients with melanoma when their pro-inflammatory effector function is measured by IFN-γ ELISPOT, while according to the literature they should be still detectable as multimer-positive, but exhausted T cells.

T cells that have TCR affinities exceeding the Level 2 TCR-L threshold will recognize MA on melanoma cells only: to them, Level-2 presentation on inflamed or regular melanocytes is subthreshold. Although such Level 3-reactive T cells recognize autoantigens that in terms of amino acid sequence are shared between tumor- and normal cells, they now become exquisitely tumor-specific due to TCR affinity-based expression level discrimination: only the > Level 2 TCR-L density on the tumor cells will meet their activation threshold. 

Unlike unvaccinated melanoma patients, we found that melanoma patients who have undergone successful AGI-101H vaccination showed strong recall responses to MA. The allogeneic IL-6 transfected melanoma cells that constitute the vaccine apparently have succeeded in triggering MA-specific T cell immunity in these subjects. Injections of these allogeneic melanoma cells in regular intervals constitutes hyperimmunization with MA. At this point, one can-not tell whether exhausted Level-2 CD8+ T cells were re-activated by the vaccine or whether Level-3-reactive CD8 cells were primed that had been naïve in MP before vaccination, or both. Possible mechanisms for the reinvigoration of exhausted CD8+ T cells include PD-1 level reduction, increased CD28 costimulation, cytokine effects, and reactivation of the stem cell-like CD8 subset which can jointly or independently reinstate effector functions of exhausted T cells (reviewed in [18]). Of these, the likelihood of the reactivation of the stem cell-like CD8 subset strikes us likely. Stem cell-like CD8+ T cells are abundant in the MA-reactive T cell repertoire of HD [21]. Stem cell–like CD8 T cells are present predominantly in the T cell zones of lymphoid tissues and are likely to continuously interact with dendritic cells in the T cell zones [18]. The AGI-101H vaccine is administered subcutaneously, delivering MA to the regional lymph nodes. The IL-6 expressed by the injected allogeneic vaccine cells, plus the local inflammatory environment triggered by allo-reactive host T cells, might contribute to the reactivation of the stem cell-like CD8+ T cells. IL-21 has been vital for sustaining CD8 T cell responses avoiding their exhaustion [36,37,38]. IL-2 has a demonstrated efficacy on melanoma [39]. While the exact mechanism of AGI-101H vaccine’s action will need to be established, the data presented here demonstrate that anti-MA immunity that went functionally dormant in unvaccinated MP has become re-engaged in the VMP, and the clinical efficacy of the vaccine suggest that these MA specific T cells might also contribute to the immune surveillance of this cancer. 

## 4. Materials and Methods

### 4.1. Human Subjects

#### 4.1.1. Healthy Controls

Peripheral blood mononuclear cells (PBMC) from healthy human donors (HD) were selected from the ePBMC library (CTL, Shaker Heights, OH, USA). The PBMC had been collected by HemaCare Blood Donor Center (Van Nuys, CA, USA) under HemaCare’s IRB and sold to CTL, identifying donors by code only while concealing the subjects’ identities. The characteristics of the randomly selected forty HD are specified in Supplemental Table S1.

#### 4.1.2. Untreated Melanoma Patients

PBMC were isolated from 24 advanced melanoma patients (MP), all in stage IV of the disease with resected or non-resected metastatic melanoma. These patients have not been treated with any immunotherapy before the blood collection. For patients with resected melanoma, sampling was performed following surgical resection of metastases, while for patients with the non-resectable disease, sampling was carried out prior to the enrollment to another treatment. Four patients underwent the surgical removal of the metastasis followed by radiotherapy completed before blood draw. All patients gave written informed consent for blood donation and PBMC collection. The clinical characteristics of these MP are summarized in Table S2.

#### 4.1.3. Melanoma Patients Treated with AGI-101H Vaccine

The AGI-101H vaccine consists of two melanoma cell lines (Mich-1 and Mich-2) that were retrovirally transduced with a fusion protein of interleukin 6 (IL-6) and its agonistic soluble IL-6 α receptor [28]. Patients with advanced melanoma, with resected or non-resected metastases, were injected subcutaneously with AGI-101H (5 × 10^7^ cells/dose) 8 times in 2-week intervals (induction phase), then every month (maintenance phase) indefinitely or until death (Clinical Trial ETAM2-5). In case of recurrence, the induction phase was repeated with or without surgery and followed by maintenance. A significant number of AGI-101H-treated patients (vaccinated melanoma patients, VMP) are still alive—out of the 138 melanoma patients enrolled in ETAM2-5 Trial, 96 (69.6%) survived a mean treatment duration of 196 months (ranging from 144 to 245 months). Twenty-three survivors from the ETAM2-5 Trial were randomly selected to participate in this study, along with four melanoma patients enrolled in the AGI-101H vaccination schedule in 2015. At the time of blood drawing, all patients treated with vaccine AGI-101H were free of clinically manifested disease. The ETAM trial was approved by the RBC and the Central Evidence of Clinical Trials (EnduraCT Number 2008-003373-40). All patients had to provide the signed informed consent for participation in the study before any study-related procedures. The clinical characteristics of the twenty-seven AGI-101H-treated melanoma patients are summarized in Supplemental Table S3.

### 4.2. Peripheral Blood Mononuclear Cell Preparation

Blood was obtained by venipuncture under sterile conditions in heparinized tubes (BD Biosciences, Franklin Lakes, NJ, USA) and PBMC were isolated within three hours by standard gradient centrifugation over Histopaque 1077 (Sigma-Aldrich, St. Louis, MO, USA). The PBMC were cryopreserved within one hour using CTL-Cryo™ ABC Media Kit (CTL) and stored in the vapor phase of liquid nitrogen until testing. Thawing, washing, and counting of PBMC were performed according to CTL protocols [40]. The PBMC were suspended at a final concentration of 2.5 million PBMC/mL in CTL-Test Medium (CTLT-005) of which 100 µL (250,000 PBMC) were plated per well into the ELISPOT assays within two hours after thawing.

### 4.3. Antigens

Five previously described [21] melanoma-associated antigens (MA) were tested in this study, each represented by a peptide pool consisting of 15-mer peptides that cover the entire amino acid sequence of the respective human protein in steps of 11 amino acids. All five MA peptide pools were purchased from JPT (Berlin, Germany): Tyrosinase (PM-Tyr), MAGE-A3 (PM-MAGEA3); melan-A/mart-1 (PM-Melan-A); Melanocyte protein Pmel 17gp100 (PM-GP100), and NY-ESO-1 (PM-NYE). All five MA peptide pools were tested at a final concentration of 1 µg/mL. As a positive control for antigen-specific T cell activation, we used CEF (from CTL, Cat #: CTL-CEF-002) that consists of a pool of 32 immune dominant nonamer peptides derived from CMV, EBV, and Flu virus [27]. The CEF pool was tested at a final concentration of 0.25 μg/mL. All peptides were dissolved in CTL-Test Medium.

### 4.4. IFN-γ ImmunoSpot Assay

Human IFN-γ single-color enzyme-linked immunospot (ELISPOT/ImmunoSpot) assays were from CTL and were performed following the manufacturer’s protocol. In brief, the PVDF membrane was pre-coated with IFN-γ capture antibody overnight. After three washes, the antigens, dissolved in CTL-Test Medium, were plated in 100 µL per well. The plates with the antigen were stored at 37 °C in a CO_2_ incubator until the cells were ready for plating. Within an hour, the freshly thawed PBMC were added at 250,000 cells/well in 100 µL using wide-bore pipette tips to avoid shear forces damaging the cells. The plates then were gently tapped on each side to ensure even sedimentation of the PBMC in the wells. The cells were incubated with the antigens for 72 h in a humidified incubator at 37 °C and 9% CO_2_. After this activation culture during which IFN-γ is captured around each antigen-stimulated T cell, the cells were removed, the IFN-γ detection antibody was added, and the plate-bound cytokine spots were visualized by enzyme-catalyzed substrate precipitation. The plates were air-dried prior to analysis. The results were analyzed using an ImmunoSpot^®^ S6 Ultimate Reader from CTL. Spot Forming Units (SFU), each representing the secretory footprint of a single IFN-γ secreting T cell, were automatically counted by the ImmunoSpot^®^ Software (CTL) that relies on statistics-based automatic setting of upper and lower size gates for SFU counting [41]. 

### 4.5. Statistical Analysis

ELISPOT counts follow normal distribution among replicate wells, which permits reliance on parametric statistics for identifying positive responses [42]. Accordingly, the Student’s *t*-test was done comparing for each test subject, and test, SFU counts in three antigen-containing replicate wells vs. the spot counts in three medium control wells. A *p*-value < 0.01 was considered as the cut-off for positivity. The non-parametric Mann-Whitney U test was performed to compare response magnitudes among cohorts, considering *p*-values < 0.01 statistically significant.

## 5. Conclusions

Natural T cell reactivity to MA detected in healthy humans is reduced in untreated melanoma patients. It is likely that during tumor growth and persisting antigen stimulation, these natural autoreactive T cells are driven into a state of exhaustion. Melanoma patients injected with allogeneic melanoma cells in form of the AGI-101H vaccine showed strong recall responses to MA. While the exact mechanism of the AGI-101H vaccine still needs to be established, the data suggest that the anti-MA immunity that went functionally dormant in untreated melanoma patients has become re-engaged in vaccinated melanoma patients.

## Data Availability

Data is contained within the article and supplementary material and are available on request from the corresponding author.

## Supplementary Materials

The following are available online at https://www.mdpi.com/2072-6694/13/2/223/s1, Figure S1: Statistical comparison of the numbers of antigen-triggered SFU counts between PBMC of healthy donors (HD), patients with untreated malignant melanoma (MP), and MP vaccinated with AGI-101H (VMP). For each subject, the IFN-γ ELISPOT recall response was tested after exposing the PBMC to the peptide pool specified for the pannels. Each data point represents the mean SFU count established in triplicate wells for a subject. The non-parametric Mann-Whitney U test was performed to reveal statistically significant differences between the specified cohorts. “ns” denotes *p* > 0.05, * *p* < 0.05, ** *p* < 0.01, and *** *p* < 0.001. Table S1: Characteristics of healthy donors (HD) tested in this study; Table S2: Characteristics of untreated melanoma patients (MP) tested in this study; Table S3: Characteristics of melanoma patients treated with the AGI-101H vaccine (VMP) tested in this study.
