# Supplementary material for: Functional T Cell Reactivity to Melanocyte Antigens Is Lost during the Progression of Malignant Melanoma, but Is Restored by Immunization
Source: Cancers (Basel). 2021 Jan 9;13(2):223. doi: 10.3390/cancers13020223 (PMC7827050; doi:10.3390/cancers13020223)
Supplement: Supplementary file 1 [file cancers-13-00223-s001.zip › Supplementary Table 1 - last.pdf]

Supplementary Table 1. Characteristics of healthy donors (HD) tested in this study.

			HLA Class I						HLA Class I		
**Donor no**	**Age**	**Gender**	**HLA - A**	**HLA - B**	**HLA - C**	**Donor no**	**Age**	**Gender**	**HLA - A**	**HLA - B**	**HLA - C**
**1**	35	Male	A*29:02	B*40:08	C*03:04	**21**	39	Female	A*03:01	B*07:02	C*02:02
			A*68:01	B*44:03	C*16:01				A*31:01	B*27:05	C*07:02
**2**	38	Male	A*30:02	B*18:01	C*04:01	**22**	34	Male	A*02:06	B*35:01	C*04:01
			A*33:03	B*53:01	C*07:01				A*03:01	B*45:01	C*06:02
**3**	24	Male	A*24:02	B*40:02	C*03:04	**23**	33	Female	A*01:01	B*13:02	C*02:02
			A*31:01	B*51:01	C*15:09				A*29:02	B*51:01	C*06:02
**4**	49	Female	A*01:01	B*14:02	C*07:01	**24**	53	Male	A*29:02	B*42:01	C*07:01
			A*01:01	B*41:02	C*17:03				A*30:01	B*49:01	C*17:01
**5**	27	Female	A*01:01	B*08:01	C*06:02	**25**	39	Male	A*02:01	B*57:04	C*06:02P
			A*68:02	B*53:01	C*07:01				A*03:01	B*58:02	C*18:02
**6**	30	Male	A*02:01	B*35:01	C*04:01	**26**	19	Female	A*01:01	B*08:01	C*04:01
			A*25:01	B*44:03	C*16:01				A*03:01	B*35:01	C*07:01
**7**	41	Male	A*02:01	B*15:01	C*04:01	**27**	36	Male	not tested	not tested	not tested
			A*23:01	B*44:03	C*04:01						
**8**	28	Male	A*02:01	B*40:01	C*03:04	**28**	22	Female	A*02:01	B*35:01	C*06:02
			A*29:02	B*44:03	C*16:01				A*03:01	B*45:01	C*16:01
**9**	41	Male	A*02:01	B*08:01	C*03:04	**29**	24	Female	A*03:01	B*35:01	C*04:01
			A*29:02	B*40:01	C*07:01				A*66:01	B*39:01	C*04:04
**10**	30	Male	A*03:01	B*15:10	C*03:04	**30**	48	Male	A*24:02	B*15:01	C*03:03
			A*24:02	B*35:01	C*04:01				A*31:01	B*18:01	C*07:01
**11**	34	Male	A*02:01	B*35:01	C*03:04	**31**	29	Female	A*11:01	B*27:05	C*01:02
			A*24:02	B*40:02	C*04:01				A*68:01	B*39:02	C*07:02
**12**	37	Male	not tested	not tested	not tested	**32**	28	Male	A*01:01	B*57:01	C*06:02
									A*02:01	B*57:01	C*06:02
**13**	51	Female	A*33:01	B*14:02	C*08:01	**33**	40	Male	not tested	not tested	not tested
			A*68:01	B*48:01	C*08:02						
**14**	25	Female	A*01:01	B*08:01	C*02:02	**34**	30	Male	A*02:01	B*39:05	C*02:02
			A*30:02	B*27:05	C*07:01				A*24:02	B*51:01	C*07:02
**15**	42	Male	A*11:01	B*35:01	C*04:01	**35**	38	Male	A*02	B*07	not tested
			A*30:02	B*44:02	C*05:01				A*30	B*35	
**16**	40	Male	A*01:01	B*08:01	C*07:01	**36**	48	Male	A*03:01	B*07:02	C*07:02
			A*29:02	B*49:01	C*07:01				A*68:02	B*14:02	C*08:02
**17**	26	Male	A*02:01	B*15:13	C*04:01	**37**	51	Male	A*01:01	B*07:02	C*07:02
			A*24:07	B*35:05	C*08:01				A*32:01	B*14:02	C*08:02
**18**	41	Male	A*11:01	B*51:01	C*03:03	**38**	51	Male	A*02:01	B*40:01	C*03:04
			A*29:02	B*51:01	C*16:01				A*02:01	B*49:01	C*07:01
**19**	26	Female	not tested	not tested	not tested	**39**	40	Male	A*01:01	B*44:03	C*06:02
									A*29:02	B*57:01	C*16:01
**20**	69	Male	A*01:01	B*08:01	C*07:01	**40**	21	Female	A*24:02	B*15:03	C*02:10
			A*01:01	B*13:02	C*07:01				A*30:01	B*40:02	C*03:04
