# Supplementary material for: Functional T Cell Reactivity to Melanocyte Antigens Is Lost during the Progression of Malignant Melanoma, but Is Restored by Immunization
Source: Cancers (Basel). 2021 Jan 9;13(2):223. doi: 10.3390/cancers13020223 (PMC7827050; doi:10.3390/cancers13020223)
Supplement: Supplementary file 1 [file cancers-13-00223-s001.zip › Supplementary Table 3 - last.pdf]

Supplementary Table 3. Characteristics of melanoma patients treated with AGI-101H (VMP) tested in this study.

Donor no	Gender	Age (at diagnosis)	Stage (at diagnosis)	Localization of primary lesion	Initial resection (primary lesion and metastasis)	Time of vaccine adm. (month)	Tumor-free period (month) *	Recurrence of melanoma	Other melanoma treatment during vaccination	Co-existing tumor
**1**	Male	52	III	torso	yes	120	120	no	no	no
**2**	Male	31	II	torso	yes	132	132	no	no	no
**3**	Female	48	III	torso	yes	132	132	no	no	no
**4**	Male	46	III	limb	yes	156	156	no	no	no
**5**	Female	38	III	limb	yes	204	204	no	no	no
**6**	Male	27	III	limb	yes	180	180	no	no	no
**7**	Female	51	III	limb	yes	120	72	yes	no **	no
**8**	Male	26	III	head	yes	120	120	no	no	no
**9**	Male	42	II	torso	yes	168	168	no	no	no
**10**	Female	33	IV	limb	yes	180	180	no	no	no
**11**	Male	50	III	torso	yes	156	156	no	no	no
**12**	Male	60	III	limb	yes	168	168	no	no	no
**13**	Female	50	III	limb	yes	180	180	no	no	no
**14**	Male	30	III	limb	yes	132	132	no	no	no
**15**	Female	26	III	limb	yes	192	192	no	no	no
**16**	Male	70	IV	limb	yes	156	156	no	no	squamous cell carcinoma ***
**17**	Male	60	III	unknown	yes	144	144	no	no	no
**18**	Female	56	III	unknown	yes	144	144	no	no	no
**19**	Male	62	III	head	yes	144	144	no	no	no
**20**	Female	41	III	limb	yes	144	144	no	no	no
**21**	Female	34	III	torso	yes	168	168	no	no	no
**22**	Male	49	IV	torso	yes	132	132	no	no	no
**23**	Female	25	III	unknown	yes	156	156	no	no	no
**24**	Female	35	III	torso	yes	60	60	no	no	no
**25**	Female	58	III	torso	yes	48	48	no	no	no
**26**	Male	78	III	head	yes	60	60	no	no	no
**27**	Female	50	II	unknown	yes	57	57	no	no	no

* disease-free survival (DSF) was defined as the time beetwen the last diagnosted and resected tumor (metastasis) and the blood sampling (PBMC isolation)
** only reinduction phase was repeated; vaccination with AGI-101H 8 times every 2 weeks
*** diagnosed in 5 month after blood sampling; not melanoma metastasis.
All patients were stable at the time of blood sampling
