# Supplementary material for: Functional T Cell Reactivity to Melanocyte Antigens Is Lost during the Progression of Malignant Melanoma, but Is Restored by Immunization
Source: Cancers (Basel). 2021 Jan 9;13(2):223. doi: 10.3390/cancers13020223 (PMC7827050; doi:10.3390/cancers13020223)
Supplement: Supplementary file 1 [file cancers-13-00223-s001.zip › SupplementaryTable 2 - last.pdf]

Supplementary Table 2. Characteristics of untreated melanoma patients (MP) tested in this study.

Donor no	Gender	Age (at diagnosis)	Stage (at diagnosis)	Localization of primary lesion	Melanoma treatment*	Melanoma proggresion**
**1**	Female	51	IV	torso	surgery, radiotherapy	yes
**2**	Male	60	IV	torso	surgery	no
**3**	Male	57	IV	torso	surgery, radiotherapy	yes
**4**	Female	63	IV	unknown	surgery	yes
**5**	Male	64	IV	head	surgery	no
**6**	Male	64	IV	limb	surgery	no
**7**	Male	35	IV	limb	surgery	yes
**8**	Male	61	IV	torso	surgery	no
**9**	Male	73	IV	torso	surgery	no
**10**	Female	45	IV	torso	surgery, radiotherapy	no
**11**	Male	65	IV	limb	surgery	yes
**12**	Male	42	IV	torso	surgery	no
**13**	Male	53	IV	torso	surgery	yes
**14**	Male	79	IV	limb	surgery	no
**15**	Female	48	IV	limb	surgery	no
**16**	Male	68	IV	torso	surgery	yes
**17**	Female	60	IV	torso	surgery	no
**18**	Female	63	IV	torso	surgery, radiotherapy	yes
**19**	Female	57	IV	head	surgery	no
**20**	Female	47	IV	limb	surgery	yes
**21**	Male	34	IV	torso	surgery	no
**22**	Female	70	IV	limb	surgery	no
**23**	Female	70	IV	limb	surgery	no
**24**	Female	54	IV	torso	surgery	yes

* treatment performed before the blood collection ; no earlier immunotherapy

** melanoma proggresion at the time of blood collection
