# Supplementary figures and images for: Functional T Cell Reactivity to Melanocyte Antigens Is Lost during the Progression of Malignant Melanoma, but Is Restored by Immunization
Source: Cancers (Basel). 2021 Jan 9;13(2):223. doi: 10.3390/cancers13020223 (PMC7827050; doi:10.3390/cancers13020223)

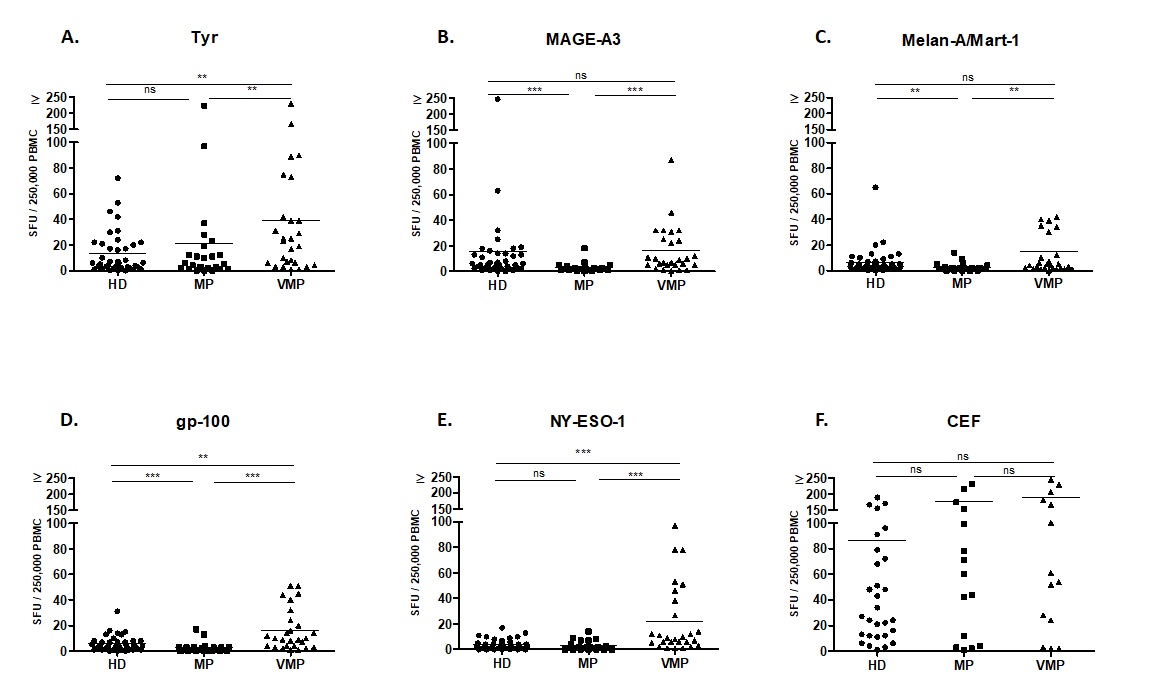

Supplement: Supplementary file 1 [file cancers-13-00223-s001.zip › Supplementary Figure 1.jpg]
